# Editorial: Challenges and future perspectives of transcatheter valve interventions

**DOI:** 10.3389/fcvm.2024.1509679

**Published:** 2024-10-28

**Authors:** Mohammad Sherif, Tobias D. Trippel, David M. Leistner

**Affiliations:** ^1^ Department of Cardiology, Loerach Hospital, Academic Teaching Hospital of the University of Freiburg, Lörrach, Germany; ^2^ Department of Cardiology, Angiology and Intensive Care Medicine, Deutsches Herzzentrum der Charité, Campus Virchow Klinikum, Berlin, Germany; ^3^ DZHK (German Centre for Cardiovascular Research), Berlin, Germany; ^4^ Department of Medicine, Cardiology, Goethe University Hospital, Frankfurt, Germany; ^5^ German Center for Cardiovascular Research (DZHK) Partner Site RheinMain, Frankfurt, Germany

**Keywords:** TAVI (transcatheter aortic valve implantation), innovation, future, perspectives, valves

**Editorial on the Research Topic**
Challenges and future perspectives of transcatheter valve interventions

## 1 Introduction: the evolution of transcatheter valve interventions

Transcatheter valve interventions (TVIs) have revolutionized the management of valvular heart disease by providing a minimally invasive option for patients at high surgical risk. This editorial synthesizes insights from six recent studies that highlight the challenges and emerging trends in TVIs, offering a comprehensive view of the current landscape and future directions in the field.

## 2 Challenges in transcatheter valve interventions

### 2.1 Patient selection and anatomical considerations

The use of a balloon-expandable valve in tricuspid valve-in-valve (ViV) procedures has shown favorable outcomes, especially for treating bioprosthetic valve failure, with improvements seen after one year of follow-up. However, this highlights the complexity of patient selection and the need to carefully consider anatomical constraints, particularly in high-risk populations Mussayev et al. Another study compared percutaneous annuloplasty and transcatheter edge-to-edge repair (T-TEER) for severe tricuspid regurgitation (TR), underscoring the challenges related to anatomical suitability and learning curves associated with each technique Mattig et al.

### 2.2 Procedural complexity and technical challenges

Addressing complications such as paravalvular leak (PVL) after initial TAVR procedures remains a significant technical challenge. A study on TAVR-in-TAVR procedures using balloon-expandable valves showed high procedural success and favorable long-term outcomes for addressing PVL, yet the complexity of these interventions underscores the need for continued device innovation Nagasaka et al. Another study on left atrial appendage occlusion (LAAO) highlighted differences in procedural complexity and outcomes when comparing single-occlusive plug-type devices to dual-occlusive disc-type devices, revealing important technical considerations that affect procedural success and complication rates Primessnig et al.

### 2.3 Post-procedural complications

Post-procedural complications such as pacemaker dependency remain a concern, particularly after TAVR with certain valve models. A comparison between the Portico and Edwards Sapien 3 valves in TAVR revealed that the Portico valve was associated with higher rates of permanent pacemaker implantation, indicating the importance of valve design in reducing complications Primessnig et al. In a study of intra-atrial shunt occlusion in older patients following embolic stroke of undetermined source, the procedure was shown to be safe and effective, although long-term outcomes in this high-risk population require further evaluation Schrader et al.

## 3 Technological innovations in TVIs

### 3.1 Advances in valve design

Technological advancements in valve design are helping to address many of the challenges described above. Balloon-expandable valves, such as those used in the tricuspid ViV and TAVR-in-TAVR procedures, have demonstrated improved procedural outcomes, reducing the need for post-procedural interventions and improving long-term valve function Mussayev et al. and Nagasaka et al. Similarly, the Edwards Sapien 3 valve has demonstrated a favorable complication profile compared to older valve models like the Portico, further highlighting the importance of valve design in optimizing outcomes Primessnig et al.

### 3.2 Hybrid approaches and new techniques

Hybrid approaches that combine percutaneous annuloplasty with T-TEER have shown potential in improving outcomes for patients with severe TR, providing a more tailored approach to complex cases Mattig et al. These hybrid strategies may expand in the future as technologies evolve and procedural techniques are refined.

## 4 Future perspectives in TVIs

### 4.1 Expanding indications

The future of TVIs lies in expanding indications beyond high-risk populations. As technology improves, TVIs are being explored for younger and lower-risk patients. For example, the successful occlusion of intra-atrial shunts in older patients with embolic stroke suggests that minimally invasive techniques can be applied safely and effectively in broader populations, including those with complex pathologies Schrader et al.

### 4.2 Personalization of valve selection and procedure planning

As seen in the comparison of the Portico and Edwards Sapien 3 valves, personalization of valve choice based on patient-specific factors will be crucial in optimizing outcomes and reducing complications Primessnig et al. This emphasis on personalized intervention will likely play a significant role in the future of TVIs, particularly as we expand the use of these technologies to more diverse patient populations.

## 5 Conclusion: A bright future for transcatheter valve interventions

TVIs continue to advance, overcoming challenges related to procedural complexity, patient selection, and device optimization. The insights from these six publications demonstrate that while hurdles remain, technological innovations are improving patient outcomes and expanding access to these life-saving procedures. The future of valve interventions is bright, driven by ongoing research into new valve designs, hybrid procedures, and personalized patient care. These advancements will likely pave the way for transcatheter interventions to become the gold standard for treating valvular disease in the years to come.

